# Assessment of Awareness and Knowledge of Proton Pump Inhibitors Among the General Population in Saudi Arabia

**DOI:** 10.7759/cureus.27149

**Published:** 2022-07-22

**Authors:** Emad S Aljahdli, Aseel M Mokhtar, Saad A Aljehani, Raad M Hamdi, Baraa H Alsubhi, Khaild F Aljuhani, Khaled A Saleh, Ammar D Alzoriri, Waleed S Alghamdi

**Affiliations:** 1 Gastrointestinal Oncology Unit, King Abdulaziz University Hospital, Jeddah, SAU; 2 Gastrointestinal Oncology Unit, King Abdulaziz University, Jeddah, SAU; 3 Medical Intern, King Abdulaziz University Faculty of Medicine, Jeddah, SAU; 4 Gastroenterology, King Abdulaziz University Hospital, Jeddah, SAU; 5 Gastroenterology, King Abdulaziz University, Jeddah, SAU

**Keywords:** proton pump inhibitors (ppis), heartburn, attitude, gastroesophageal reflux, saudi arabia, adverse effects, behavior, proton pump inhibitor, antacids, awareness

## Abstract

Background

One of the most commonly used classes of medications that are known for their excessively expanded misuse is proton pump inhibitors (PPIs). Although they are profoundly useful, they also account for several adverse effects. Assessing the awareness of the general population may throw light on the problem and limit irrational use. This study aims to determine the knowledge, attitude, and behavior of the general population of Saudi Arabia regarding PPI use.

Materials and methods

This was a descriptive cross-sectional study using a structured online survey. The questionnaire comprised 1088 participants of the adult general population of Saudi Arabia to assess knowledge and awareness of proton pump inhibitors.

Results

Of the 1088 participants, 59% were men and 41% were women, 44.6% were aged 20-30 years, 25.4% were 31-40 years, and 16% were 41-50 years. Only 54% of the participants had previous knowledge of PPIs. Regarding PPI use, 25.7% of participants previously used PPIs with medical consultation and 10.3% without medical consultation, while 64% had never used PPIs. Reasons for PPI use included: heartburn (56.4%), gastroesophageal reflux disease (51.1%), gastritis (21.8%), as part of *Helicobacter pylori* infection therapy (20%), peptic ulcer (15.7%), esophagitis (7.1%), sore throat (6.1%), gastroenteritis (5.4%), hiatal hernia (5%), hoarseness (3.2%), asthma (2.1%), and bariatric surgery (2.1%). Of all the participants, 61.2% completed the treatment course while 38.8% did not.

Conclusion

Generally, our population had moderate knowledge of PPI. However, it is not enough to eliminate this irrational use of PPIs.This study emphasizes the importance of effective provider-led patient education to raise awareness of potential risks and reduce inappropriate long-term use of PPIs. This is significant due to growing concern about the possibility of medication overuse and non-compliance due to a lack of awareness about PPIs. In addition, more research assessing the awareness of over-the-counter (OTC) medications should be taken into consideration.

## Introduction

Proton pump inhibitors (PPIs) are among the most commonly prescribed class of drugs globally [1]. Between 2002 and 2009, the archived use of PPIs increased by nearly 10-fold in the United States, where it is among the top 10 most prescribed drugs [2]. Inappropriate PPI prescriptions are considered a financial burden for the government and the general population costing approximately £2 billion per year, globally. PPIs function by decreasing acid secretion in the stomach through their effect on parietal cells, inhibiting the hydrogen-potassium ATPase pump, which decreases acid secretion [3]. PPIs increase gastric pH, which might encourage the growth of gut microflora, increase bacterial translocation, and alter various immunomodulatory and anti-inflammatory effects [4]. The long-term use of PPIs for treatment has increased as they can be obtained over the counter and outside health care facilities, and several studies have reported excessive use of PPIs that exceeds the number of reported cases with gastrointestinal symptoms. Although PPIs may be effective, these factors have resulted in widespread inappropriate PPI use. In an academic hospital in Saudi Arabia, a study by Basheikh and his colleagues reported a prevalence rate of 57.6% for PPI prescriptions [5]. PPIs play an indispensable role in treating peptic ulcer diseases (PUDs), gastroesophageal reflux disease (GERD), Helicobacter pylori infection, and dyspepsia, and have been shown to prevent stress ulcers in patients using non-steroidal anti-inflammatory drugs (NSAIDs) [6]. Similar to other medications, PPIs cause several side effects, such as vitamin B12 deficiency, bone fracture, and gastrointestinal infections [7]. Even when PPIs are well tolerated, many recent studies have reported their associations with adverse effects [8]. According to some studies, patient awareness of the adverse effects associated with PPIs is very low, showing that most participants were unfamiliar with any adverse effects associated with PPI use [9-10]. Therefore, this study aimed to determine the awareness, attitude, and behavior of the general population of Saudi Arabia regarding PPIs through a cross-sectional study design using self-administered questionnaires.

## Materials and methods

Study design, setting, and participants

This descriptive cross-sectional study is a structured online survey. The sample size was determined using Raosoft, with the settings at a minimum sample size of 385, at 95% confidence, and 5% error. The total number of participants that took part in the survey was 1088. The participants who were under 18 years of age were excluded.

Data collection

A structured online survey on the awareness, knowledge, and behavior of participants regarding proton pump inhibitors (PPIs) was designed and data was collected over two weeks, from August 12 to August 28, 2021. Five content experts validated the draft survey, and minor modifications were made based on their suggestions. The questionnaire had three sections. The first section included the obtained consent to participate in this research. The second section contains the collected social and demographic data on seven to eight items, including age, sex, nationality, region, marital status, level of education, and whether the participant worked in the medical field (if the answer was yes, the participant specified the exact occupation from a single answer multiple-choice question). The third section included the measurements of participants' awareness, knowledge, and behavior regarding PPIs through eight single-response multiple-choice items. The content areas of the survey were "knowledge and indications of using PPIs" (three items; items 1, 2, and 3), "behavior and usage" (four items; items 4, 5, 6, and 7), and "knowledge of common side effects" (item 8). 

Procedure for data collection

Following ethical approval, the online link to the questionnaire, which was created using Google Forms, was sent via social media. Participation was optional. Participants were informed about the study objectives and then answered the demographic and knowledge questions. Participants took three to four minutes on average to complete the questionnaire.

Ethical consideration

Ethical approval was obtained from the Institutional Review Board (IRB) of King Abdul-Aziz University Hospital, Jeddah, Saudi Arabia, following the Declaration of Helsinki guidelines (Ref. No. 407-21). All data were kept confidential and were available only to the research team.

Statistical analysis

Statistical analysis was performed using Statistical Package for Social Sciences (SPSS) version 23 (IBM Corp., Armonk, NY, USA). Data are presented as means and standard deviations (SD). For categorical data, absolute and relative frequencies were used to determine the difference in knowledge scores between the two groups. The chi-square test was used to determine the association between sociodemographic factors and knowledge scores and the use of PPIs. Using Student’s t-test, significance thresholds were established at P < 0.05.

## Results

Of the 1088 participants in our study, 59% were men and 41% were women. Regarding age, 44.6% of participants were between 20-30 years, 25.4% were between 31-40 years, and 16% were between 41-50 years. Of the studied samples, 90.6% were from Saudi Arabia. A total of 51.5% of participants were married and 45.2% were single. Only 26% of the participants worked in the medical field (13.1% as students, 4.7% as doctors, 2.8% as administrators, 1.9% as pharmacists, 0.8% as nurses, 1.7% as technicians, and 1% as others). Regarding educational level, 52.5% of participants had a bachelor’s degree and 26.2% had a high school certificate. Table 1 illustrates sociodemographic characteristics of participants.

**Table 1:** Sociodemographic characteristics of participants

Parameter		Number (n=1088)	Percentage %
Gender	Male	642	59
	Female	446	41
Age	Less than 20	62	5.7
	20 - 30	485	44.6
	31 - 40	276	25.4
	41 - 50	174	16
	51 - 60	71	6.5
	More than 60	20	1.8
Nationality	Saudi	986	90.6
	Non-Saudi	102	9.4
Marital status	Married	560	51.5
	Single	492	45.2
	Divorced	23	2.1
	Widow	13	1.2
Region	Western	752	69.1
	Southern	87	8
	Eastern	53	4.9
	Northern	37	3.4
	Middle	159	14.6
Work in the medical field	Yes	283	26
	No	805	74
If yes, field	Student	142	13.1
	Doctor	51	4.7
	Pharmacist	21	1.9
	Nurse	9	0.8
	Technician	18	1.7
	Administrator	31	2.8
	Other	11	1
	Missing	805	74
Education level	Less than high school	39	3.6
	High school	285	26.2
	Bachelor	571	52.5
	Higher degree	103	9.5
	Diploma	90	8.3

Only 54% of the participants had previously heard of PPIs. Regarding PPI use, 25.7% of participants previously used PPIs after medical consultation, and 10.3% used them without medical consultation. Of all uses, 15.7% were for peptic ulcer, 51.1% for gastroesophageal reflux disease, 56.4% for heartburn, 20% for *Helicobacter pylori* infection, 7.1% for esophagitis, 5% for hiatal hernia, 2.1% for asthma, 3.2% for hoarseness, 21.8% for gastritis, 5.4% for gastroenteritis, 6.1% for sore throat, and 2.1% after bariatric surgery. Of all participants, 64% never used PPIs. Table 2 illustrates knowledge of participants of PPI.

**Table 2:** Knowledge of participants of proton pump inhibitors (PPIs) Participants who used PPIs can choose more than one indication at the same time.

Parameter		Number (n=1088)	Percentage %
Heard of PPI	Yes	588	54
	No	500	46
Used PPI before	Yes, by medical consultation	280	25.7
	Yes, without medical consultation	112	10.3
	I have never used it	696	64
If yes, Indication (n=392)	Peptic ulcer	44	15.7
	Gastroesophageal reflux disease	143	51.1
	Heartburn	158	56.4
	Helicobacter pylori infection	56	20
	Esophagitis	20	7.1
	Hiatal hernia	14	5
	Asthma	6	2.1
	Hoarseness	9	3.2
	Gastritis	61	21.8
	Gastroenteritis	15	5.4
	Sore throat	17	6.1
	After bariatric surgery	6	2.1

For the duration of use, 76.8% (301 participants) of those who used PPIs were using them as needed, 11.6% (46 participants) were not regular, 4.5% (17 participants) were regular for more than four weeks, 3.6% (14 participants) were regular for less than four weeks, and 3.6% who used the medication did not continue for the recommended duration of the treatment. The appropriate time to take PPIs was reported to be before meals by 59.8% of participants, after meals by 39.3%, and with meals by 0.9%. Of all the participants who used PPIs, only 62.2% completed the course of treatment. Among those who did not complete the course, 71.7% reported that it was due to symptom improvement, 13.1% due to fear of side effects, 5.2% due to their side effects, 3.9% due to advice from friends or relatives in the medical field, 2.6% because the drug was unaffordable or unavailable, and in 1.3% owing to advise from friends or relatives outside the medical field. Table 3 illustrates the duration of using the drug and course completion among participants who used PPIs.

**Table 3:** Duration of using the drug and course completion among participants who used proton pump inhibitors (PPIs)

Parameter		Number (n=392)	Percentage %
Duration of using PPI if used	As needed	301	76.8
	Regularly more than 4 weeks	17	4.5
	Regularly less than 4 weeks	14	3.6
	Not regularly	46	11.6
	Used the medication but did not complete the recommended duration	14	3.6
Appropriate time to take PPI	Before meal	234	59.8
	After meal	154	39.3
	With meal	4	0.9
Completed the course, if used PPI	Yes	240	61.2
	No	152	38.8
If no, why (n=152)	Due to side effects	8	5.2
	Fear of side effects	20	13.1
	Not affordable, not available	4	2.6
	Symptom’s disappearance, no longer needed	109	71.7
	Advice from a friend or relative (in the medical field)	6	3.9
	Advice from a friend or relative (outside the medical field)	2	1.3
	Missed	3	1.9

Results of this study showed significant relationship between participants of older age and knowledge of PPIs more frequently in comparison with younger age participants (p-value=0.001). Moreover, working in the medical field is associated with higher knowledge level (p-value=0.001). Unsurprisingly, there is significant association between higher education level and hearing of PPIs, as 58% of those with a Bachelor degree have heard about PPIs (p-value=0.001). We did not find an association in gender category as we had expected, as regurgitation is common during pregnancy and increased BMI (p-value=0.905). Table 4 illustrates association between participants’ knowledge of PPIs with sociodemographic characteristics of participants.

**Table 4:** Association between participants’ knowledge of proton pump inhibitors (PPIs) with sociodemographic characteristics of participants

Variables		Heard of PPIs		Total (n=1088)	P value
		Yes	No		
Gender	Male	346	296	642	0.905
		53.90%	46.10%	59.00%	
	Female	242	204	446	
		54.30%	45.70%	41.00%	
Age	Less than 20	14	48	62	0.001
		22.60%	77.40%	5.70%	
	20 - 30	263	222	485	
		54.20%	45.80%	44.60%	
	31 - 40	150	126	276	
		54.30%	45.70%	25.40%	
	41 - 50	103	71	174	
		59.20%	40.80%	16.00%	
	51 - 60	46	25	71	
		64.70%	35.30%	6.50%	
	More than 60	12	8	20	
		60.00%	40%	1.80%	
Nationality	Saudi	541	445	986	0.09
		54.90%	45.10%	90.60%	
	Non-Saudi	47	55	102	
		46.10%	53.90%	9.40%	
Marital status	Married	295	265	560	0.07
		52.70%	47.30%	51.50%	
	Single	266	226	492	
		54.10%	45.90%	45.20%	
	Divorced	18	5	23	
		78.30%	21.70%	2.10%	
	Widow	9	4	13	
		69.20%	30.80%	1.20%	
Region	Western	376	376	752	0.002
		50.00%	50.00%	69.10%	
	Southern	55	32	87	
		63.20%	36.80%	8.00%	
	Eastern	32	21	53	
		60.40%	39.60%	4.90%	
	Northern	22	15	37	
		59.50%	40.50%	3.40%	
	Middle	103	56	159	
		64.80%	35.20%	14.60%	
Work in Medical field	Yes	234	49	283	0.001
		82.70%	17.30%	26.00%	
	No	354	451	805	
		44.00%	56.00%	74.00%	
If the answer is yes, then what field do you belong to	Health faculties student	121	21	142	0.001
		85.20%	14.80%	13.10%	
	Doctor	48	3	51	
		94.10%	5.90%	4.70%	
	Pharmacist	20	1	21	
		95.20%	4.80%	1.90%	
	Nurse	9	0	9	
		100%	0.00%	0.80%	
	Technician	8	10	18	
		44.40%	55.60%	1.70%	
	Administrator	18	13	31	
		58.10%	41.90%	2.80%	
	Other	10	1	11	
		1.70%	0.20%	1.00%	
	Missing	354	451	805	
		90.10%	9.90%	74.00%	
Education level	Less than high school	10	29	39	0.001
		25.60%	74.40%	3.60%	
	High school	140	145	285	
		49.10%	50.90%	26.20%	
	Bachelor	331	240	571	
		58.00%	42.00%	52.50%	
	Higher degree	61	42	103	
		59.20%	40.80%	9.50%	
	Diploma	46	44	90	
		51.10%	48.90%	8.30%	

Results of this study in general showed significant relationship between participants of older age and the increased use of PPIs in comparison with younger age participants (p-value=0.001). Furthermore, working in the medical field was associated with more frequent use of PPIs without medical consultation (p-value=0.001). Males tend to use PPIs without medical consultation (12.60%) more than females (7.00%) (p-value=0.001). Table 5 illustrates the association between participants’ use of PPIs with sociodemographic characteristics.

**Table 5:** Association between participants’ use of proton pump inhibitors (PPIs) with sociodemographic characteristics

Variables		Used PPIs			Total	P value
		Yes, by medical consultation	Yes, without medical consultation	I have never used it		
Gender (n=1088)	Male	159	81	402	642	0.01
		24.70%	12.60%	62.60%	59.00%	
	Female	121	31	294	446	
		27.10%	7.00%	65.90%	41.00%	
Age (n=1088)	Less than 20	5	1	56	62	0.001
		8.10%	1.60%	90.30%	5.70%	
	20 - 30	77	41	367	485	
		15.90%	8.50%	75.70%	44.60%	
	31 - 40	94	33	149	276	
		34.10%	12.00%	54.00%	25.40%	
	41 - 50	64	26	84	174	
		36.80%	14.90%	48.30%	16.00%	
	51 - 60	31	10	30	71	
		43.70%	14.10%	42.30%	6.50%	
	More than 60	9	1	10	20	
		45.00%	5.00%	50.00%	1.80%	
Nationality (n=1088)	Saudi	254	102	630	986	0.981
		25.80%	10.30%	63.90%	90.60%	
	Non-Saudi	26	10	66	102	
		25.50%	9.80%	64.70%	9.40%	
Marital status (n=1088)	Married	168	66	326	560	0.001
		30.00%	11.80%	58.20%	51.50%	
	Single	91	40	361	492	
		18.50%	8.10%	73.40%	45.20%	
	Divorced	16	3	4	23	
		69.60%	13.00%	17.40%	2.10%	
	Widow	5	3	5	13	
		38.50%	23.10%	38.50%	1.20%	
Region (n=1088)	Western	175	70	507	752	0.005
		23.30%	9.30%	67.40%	69.10%	
	Southern	25	6	56	87	
		28.70%	6.90%	64.40%	8.00%	
	Eastern	21	7	25	53	
		39.60%	13.20%	47.20%	4.90%	
	Northern	11	8	18	37	
		29.70%	21.60%	48.60%	3.40%	
	Middle	48	21	90	159	
		30.20%	13.20%	56.60%	14.60%	
Have you ever worked in the medical field? (n=1088)	Yes	77	44	162	283	0.001
		27.20%	15.60%	57.20%	26.00%	
	No	203	68	534	805	
		25.20%	8.40%	66.30%	74.00%	
If the answer is yes, then what field do you belong to? (n=162)	Health faculties student	26	15	101	142	0.001
		18.30%	10.60%	71.10%	13.10%	
	Doctor	17	17	17	51	
		33.30%	33.30%	33.30%	4.70%	
	Pharmacist	8	6	7	21	
		38.10%	28.60%	33.30%	1.90%	
	Nurse	5	1	3	9	
		55.60%	11.10%	33.30%	0.80%	
	Technician	3	3	12	18	
		16.70%	16.70%	66.70%	1.70%	
	Administrator	12	2	17	31	
		38.70%	6.50%	54.80%	2.80%	
	Other	6	0	5	11	
		54.50%	0.00%	45.50%	1.00%	
	Missing	203	68	534	805	
		25.20%	8.40%	66.30%	74.00%	
Education level (n=1088)	Less than high school	8	1	30	39	0.001
		20.50%	2.60%	76.90%	3.60%	
	High school	63	18	204	285	
		22.10%	6.30%	71.60%	26.20%	
	Bachelor	147	62	362	571	
		25.70%	10.90%	63.40%	52.50%	
	Higher degree	34	24	45	103	
		33.00%	23.30%	43.70%	9.50%	
	Diploma	28	7	55	90	
		31.10%	7.80%	61.10%	8.30%	

Individuals who had used PPIs previously reported nausea (27.6%) followed by bloating and diarrhea (22.4%, 17.9% respectively) as the most common side effects. Those who had never used PPIs reported bloating, abdominal pain, and nausea as common side effects (34.8%, 20.4%, and 18% respectively). Blockage of heart stent appeared to be the least recognizable side effect of PPIs among both those who had and those who had not used PPIs (1.5% and 1.6%, respectively). Table 6 illustrates participants’ use and knowledge of PPI side effects.

**Table 6:** Participants’ use and knowledge of proton pump inhibitors (PPIs) side effects

Knowledge about side effects (n=1088)			
				Side effect	Answer	Used Medication	Didn’t use Medication
Headache	Yes	16.60%	16.50%
	No	83.40%	83.50%
Bloating	Yes	22.40%	34.80%
	No	77.60%	65.20%
Nausea	Yes	27.60%	18.00%
	No	72.40%	82.00%
Abdominal pain	Yes	17.10%	20.40%
	No	82.90%	79.60%
Diarrhea	Yes	17.90%	16.70%
	No	82.10%	82.30%
Constipation	Yes	14.00%	11.40%
	No	86.00%	88.60%
Deterioration of kidney function ¶	Yes	13.50%	7.80%
	No	86.50%	92.20%
Blockage of heart stent ¶	Yes	1.50%	1.60%
	No	89.50%	98.40%
Osteoprosis	Yes	14.50%	4.50%
	No	85.50%	95.50%
Demantia	Yes	3.10%	1.90%
	No	96.90%	98.10%
Increase risk of fracture	Yes	8.70%	3.20%
	No	91.30%	96.80%
Iron deficiency anemia	Yes	12.20%	3.90%
	No	87.80%	96.10%
Mineral deficiency	Yes	14.50%	6.80%
	No	85.50%	93.20%
Increase risk of gastroenteritis	Yes	5.40%	5.30%
	No	94.60%	94.70%
Gastric cancer ¶	Yes	6.60%	2.40%
	No	93.40%	97.60%
¶ Hypothesized yet unproven side effects that are linked to PPIs.			

## Discussion

PPIs are among the most frequently prescribed drugs globally, and the wide availability of OTC PPIs has contributed to their widespread use. In recent years, their popularity has risen [11]. Several studies have found that the use of and expenditure on PPIs have increased six to 10 times in the last decade [12]. Irrational PPI use has been shown to have adverse therapeutic outcomes. Interestingly, our study showed that 28.5% of those who used PPIs obtained them without a prescription. Previous studies have revealed that PPI abuse can lead to many side effects, including bone fractures, mineral and nutrient deficiencies, and *C. difficile* infection. Other secondary diseases identified in patients who have used PPIs for a long time include dementia progression, pneumonia, gastric cancer, and chronic kidney disease [13]. Our study assessed participants’ knowledge about the common side effects of PPIs and unproven side effects that are linked to PPIs and concern most patients, including gastric cancer, blockage of heart stents, and deterioration of kidney function. Notably, these side effects are hypothesized and not yet proven, and those who believe gastric cancer to be a side effect of the long-term use of PPIs might discontinue the medication. Our results generally showed poor recognition of PPIs' side effects, emphasizing the doctor's role in educating patients.

Only 54% of the participants in our study were previously aware of PPIs. In terms of PPI use, 25.7% used PPIs with medical consultation and 10.3% without medical consultation, while 64% had never used PPIs. In comparison, a Lebanese study found that 71.4% of the study population overused PPIs [14]. Another study found that inappropriate PPI use ranged from 40% to 81% with a mean of 63% [15]. Rotman et al. assessed PPI use in an ambulatory setting in the United States and found that 62.9% of PPI users had no documented gastrointestinal diagnoses/complaints or other appropriate indications [16]. Our study showed that only 3.6% of participants who used PPIs did not know their indication of use. Further research performed by Ntaios et al. found that PPIs were taken by 25.4% of inpatients in a Greek tertiary hospital, but 81.2% of these patients had no indications or instructions regarding the duration of treatment after discharge [17]. PPIs are currently the most effective inhibitors of acid secretion. They have become the treatment of choice for a wide range of acid-related gastrointestinal disorders, and the perspective on these diseases has shifted dramatically. These medications have virtually eliminated elective peptic ulcer surgery and significantly reduced the mortality and morbidity associated with stress-related ulcers and NSAID-induced gastropathy [3,10] The indications for PPI use in our study were heartburn (56.4%), gastroesophageal reflux disease (51.1%), gastritis (21.8%), *H. pylori* infection (20%), peptic ulcer (15.7%), esophagitis (7.1%), sore throat (6.1%), gastroenteritis (5.4%), hiatal hernia (5%), hoarseness (3.2%), asthma (2.1 %), and bariatric surgery (2.1%). Our study is consistent with previous studies that showed that GERD/heartburn constituted the majority of indications for PPI prescriptions [18,19]. For example, the participants in another study were mainly prescribed PPIs by gastroenterologists [9]. Another study reported that most participants were taking PPIs for gastroprotection [15]. However, insufficient instructions regarding treatment duration may result in overuse. Our study showed that the most common cause of medication discontinuation was symptom disappearance (71.7%); other causes included fear of side effects (13.1%) and the presence of side effects (5.2%). Another study reported various reasons for PPI discontinuation, including provider's instructions, failure of medication to achieve relief, affordability, and the decision to switch to a different therapy [9]. In our study, 76.8% of patients who used PPIs reported that the duration of use was as needed, 11.6% were not regular, 4.5% were regular for more than four weeks, 3.6% were regular for less than four weeks, and 3.6% used the medication but did not continue for the recommended duration. In addition, 24.1% of participants who used PPIs completed the course of treatment. Among those who did not complete the course, 71.7% were due to symptom disappearance, 13.1% due to fear of side effects, 5.2% due to side effects, 3.9% due to advice from a friend or relative in the medical field, 2.6% because the drug was not affordable or not available, 1.9% had missing data, and 1.3% due to advice from a friend or relative outside the medical field. According to one study, 22.1% of participants took PPIs for medically approved reasons but for a longer period than indicated. Whether these patients were followed up by their doctors to evaluate the need for continued therapy is unknown [14]. According to a study conducted by Reimer and Bitzer, only 27% of patients receiving PPIs on a long-term basis had a diagnosis that justified the need for long-term therapy [20]. Our study found a significant association between knowledge of PPI and participant age, working in the medical field, and educational level. In addition, a significant association was noted between previous use of PPIs and sex, age, marital status, work in the medical field, and educational level of participants. Notably, this study found no differences in medication compliance among participants of different ages. White and his colleagues also described a lack of risk perception regarding PPIs in a population with a high poverty rate and low educational attainment [9]. A different study found that 63% of patients had a high school diploma or lower.

Because of the extent of long-term and high-dose PPI use observed among patients with low educational levels and socioeconomic status [20,21], the low awareness of reported adverse effects associated with PPIs in this population is especially relevant. This is consistent with our study that showed that patients with low education levels were more subject to adverse effects since 43.6% of them did not know about the side effects and had limited knowledge of PPIs, as demonstrated by only 23.7% having previously heard about PPIs. Differences in patient perceptions of risks associated with PPIs were observed in studies by Ghosh et al. and Kurlander et al. [19,22] and may also be explained by differences in the socio-economic and educational characteristics of the study population [19,22]. Another study found that 45% of the surveyed patients were aware of the adverse effects of PPIs, but this rate was significantly lower among patients who did not complete high school [19]. Healthcare providers can reassure patients about the safety of OTC products in conjunction with prescription medications and discuss potential opportunities to optimize acid-related disease management through pharmacologically sound therapeutic combinations. Furthermore, there is unquestionably a significant underutilization of corrective lifestyle changes that can help with reflux symptoms (e.g., avoiding bedtime snacking and tobacco use and various dietary adjustments, including weight loss). When used correctly, individualized therapy can significantly improve patients' symptoms of GERD [23].

Limitation

This questionnaire was appropriated electronically, influenced by psychological and surrounding components. We cannot generalize the results because the responders were mostly from the western region of Saudi Arabia.

## Conclusions

Generally, our population had moderate knowledge of PPIs. However, it is not enough to eliminate this irrational use of PPIs. This study emphasizes the importance of effective provider-led patient education to raise awareness of potential risks and reduce inappropriate long-term use of PPIs. This is significant due to growing concern about the possibility of medication overuse and non-compliance due to a lack of awareness about PPIs. In addition, more research assessing the awareness of OTC medications should be taken into consideration.
